# Differential Diagnosis of Thyrotoxicosis by Machine Learning Models with Laboratory Findings

**DOI:** 10.3390/diagnostics12061468

**Published:** 2022-06-15

**Authors:** Jinyoung Kim, Han-Sang Baek, Jeonghoon Ha, Mee Kyoung Kim, Hyuk-Sang Kwon, Ki-Ho Song, Dong-Jun Lim, Ki-Hyun Baek

**Affiliations:** 1 Division of Endocrinology and Metabolism, Department of Internal Medicine, Yeouido St. Mary’s Hospital, College of Medicine, The Catholic University of Korea, 10 63-ro, Yeongdeungpo-gu, Seoul 07345, Korea; julia@catholic.ac.kr (J.K.); makung@catholic.ac.kr (M.K.K.); drkwon@catholic.ac.kr (H.-S.K.); kihos@catholic.ac.kr (K.-H.S.); 2 Division of Endocrinology and Metabolism, Department of Internal Medicine, Seoul St. Mary’s Hospital, College of Medicine, The Catholic University of Korea, 222 Banpo-daero, Seocho-gu, Seoul 06591, Korea; hsbaek1209@catholic.ac.kr (H.-S.B.); 3002041@catholic.ac.kr (J.H.); ldj6026@catholic.ac.kr (D.-J.L.)

**Keywords:** thyrotoxicosis, hyperthyroidism, differential diagnosis, machine learning

## Abstract

Differential diagnosis of thyrotoxicosis is essential because therapeutic approaches differ based on disease etiology. We aimed to perform differential diagnosis of thyrotoxicosis using machine learning algorithms with initial laboratory findings. This is a retrospective study through medical records. Patients who visited a single hospital for thyrotoxicosis from June 2016 to December 2021 were enrolled. In total, 230 subjects were analyzed: 124 (52.6%) patients had Graves’ disease, 65 (28.3%) suffered from painless thyroiditis, and 41 (17.8%) were diagnosed with subacute thyroiditis. In consideration that results for the thyroid autoantibody test cannot be immediately confirmed, two different models were devised: Model 1 included triiodothyronine (T3), free thyroxine (FT4), T3 to FT4 ratio, erythrocyte sediment rate, and C-reactive protein (CRP); and Model 2 included all Model 1 variables as well as thyroid autoantibody test results, including thyrotropin binding inhibitory immunoglobulin (TBII), thyroid-stimulating immunoglobulin, anti-thyroid peroxidase antibody, and anti-thyroglobulin antibody (TgAb). Differential diagnosis accuracy was calculated using seven machine learning algorithms. In the initial blood test, Graves’ disease was characterized by increased thyroid hormone levels and subacute thyroiditis showing elevated inflammatory markers. The diagnostic accuracy of Model 1 was 65–70%, and Model 2 accuracy was 78–90%. The random forest model had the highest classification accuracy. The significant variables were CRP and T3 in Model 1 and TBII, CRP, and TgAb in Model 2. We suggest monitoring the initial T3 and CRP levels with subsequent confirmation of TBII and TgAb in the differential diagnosis of thyrotoxicosis.

## 1. Introduction

Differential diagnosis of thyrotoxicosis is essential because therapeutic approaches differ based on disease etiology [1]. The most common cause of thyrotoxicosis is Graves’ disease, an autoimmune disease characterized by increased thyroid hormone synthesis. The standard treatment for Graves’ disease is thioamide-based drugs, a group of anti-thyroid drugs (ATD) that concentrates in the thyroid gland and inhibits hormone synthesis [2]. Other common causes include painless thyroiditis due to autoimmune lymphocytic infiltration [3], and subacute thyroiditis due to viral infection [4]. In these cases, thyrotoxicosis caused by thyroid tissue destruction is transient and management is symptomatic therapy with cautionary observation.

Finding the etiology of thyrotoxicosis can be challenging in patients experiencing their first presentation of thyrotoxicosis, and the etiology of thyrotoxicosis is sometimes revealed after considerable clinical progression. For Graves’ disease, the diagnostic accuracy of thyroid stimulating hormone (TSH) receptor antibodies has been improved [5], but autoantibody tests require several days to obtain results and can have inconsistent or low accuracy depending on type and generation [6]. Thyroid scintigraphy and sonography may be used as adjuncts, but there are several pitfalls. Thyroid scans have the disadvantage of not being accessible in primary care clinics, yielding false negative results in patients on a high iodine diet or taking ATD [7]. It is also contraindicated in pregnant or lactating women. Doppler ultrasonography can show hyper-vascularization of the thyroid that is similar to Graves’ disease even in hypothyroidism, which can make diagnosis difficult [8].

Therefore, we aimed to perform differential diagnoses of thyrotoxicosis using a machine learning algorithm with initial laboratory findings, including triiodothyronine (T3), free thyroxine (FT4), T3 to FT4 ratio, erythrocyte sediment rate (ESR), C-reactive protein (CRP), and the results of thyroid autoantibodies-thyrotropin binding inhibitory immunoglobulin (TBII), thyroid-stimulating immunoglobulin (TSI), anti-thyroid peroxidase antibody (TPOAb), and anti-thyroglobulin antibody (TgAb).

## 2. Materials and Methods

### 2.1. Patients

Patients who visited endocrinology clinics at a single hospital for thyrotoxicosis—defined as high free thyroxine (FT4) and low thyroid stimulating hormone (TSH) levels beyond the reference ranges—from June 2016 to December 2021 were enrolled. The final diagnosis was confirmed with retrospective review of the clinical course over at least six months by two physicians (K.-H.B.; 20 years of experience in endocrinology, and J.K.; 3 years of experience in endocrinology). Patients with a previous history of thyroid disease (*n* = 70), hyper-functioning nodule (*n* = 5), and insufficient test results (*n* = 12) were excluded. The protocol of this study was approved by the Institutional Review Board of Yeouido St. Mary’s Hospital (SC21OISI0070). Consent from each patient was waived as this clinical study is a retrospective review of medical records produced during the patient’s treatment process.

### 2.2. Measurements

We collected the results of initial laboratory findings including T3, FT4, TSH, ESR, CRP, TBII, TSI, TPOAb, and TgAb at the first visit. For thyroid function, TSH, T3, and FT4 levels were performed using the Elecsys Cobas kit (Roche Diagnostics International Ltd., Rotkreuz, Switzerland), and the normal ranges were 0.27−4.2 μIU/mL, 0.8−2.0 ng/mL, and 0.93−1.7 ng/dL, respectively. For quantitative evaluation of TSH-receptor antibodies in study subjects, TBII and TSI were measured with a commercial kit using second generation methods. TBII was measured using TRAK human radioimmunoassay (Thermo Scientific, Waltham, MA, USA), and 1 IU/L was used as the cut-off according to the manual. TSI was measured using the Thyretain TSI Reporter Bioassay (Diagnostic Hybrids, Athens, OH, USA), and 140% was the cut-off. TPOAb and TgAb titers were measured with the Elecsys Cobas kit (Roche Diagnostics). For quantitative CRP measurements, the Tina-Quant kit (Roche Diagnostics) was used with a cut-off of 5.0 mg/L, and ESR was measured with the Test 1 automated analyzer (Alere Healthcare, Seoul, Korea), with a cut-off of 20 mm/h.

### 2.3. Clinical Validation

To compare between the standard diagnostic approach and machine learning algorithms, we collect the results of thyroid scans and the prescriptions of ATD by the patients’ clinicians during the initial presentation through a retrospective review. These results were compared with the diagnostic accuracy of Graves’ disease of our machine learning models.

### 2.4. Statistical Analyses

For descriptive statistics, continuous variables were expressed as mean (standard deviation), and categorical variables were expressed as number (percentage), and analysis of variance and chi-square tests were used for comparison between groups. In consideration that thyroid autoantibody test results cannot be immediately confirmed, two major analyses were performed: Model 1 analyzed patient T3, FT4, FT4 to T3 ratio, ESR, and CRP; and Model 2 added TBII, TSI, TPOAb, and TgAb to the Model 1 variables. Differential diagnosis accuracy was calculated using seven machine learning algorithms—classification and regression tree, random forest, linear discriminant analysis, support vector machine, k-nearest neighbor, naive Bayesian, and neural network. Before the analysis was performed, all data were randomly divided into 7:3 ratio and 30% of subjects were used as the test set.

Statistical analyses were performed in R version 4.0.5. For the classification and regression tree analysis (CART), the ‘ctree’ function of the ‘party’ package was used. Random forest analysis (RF) was performed using the ‘randomForest’ function of ‘randomForest’ package, and ‘varlmpPlot’ functions were used to estimate the prediction significance of each variable. For linear discriminant analysis (LDA), the ‘lda’ function of the ‘MASS’ package was used, and for the support vector machine (SVM), the ‘svm’ function of the ‘e1071′ package was used. To calculate k-nearest neighbor (kNN), the ‘knn’ function of the ‘class’ package was used, and the ‘e1071′ package with the ‘naiveBayes’ function was used to evaluate the naive Bayesian (NB). For the neural network (NN), the ‘nnet’ function of the ‘nnet’ package was used.

## 3. Results and Discussion

### 3.1. Baseline Characteristics of the Study Cohort

**Figure 1:**
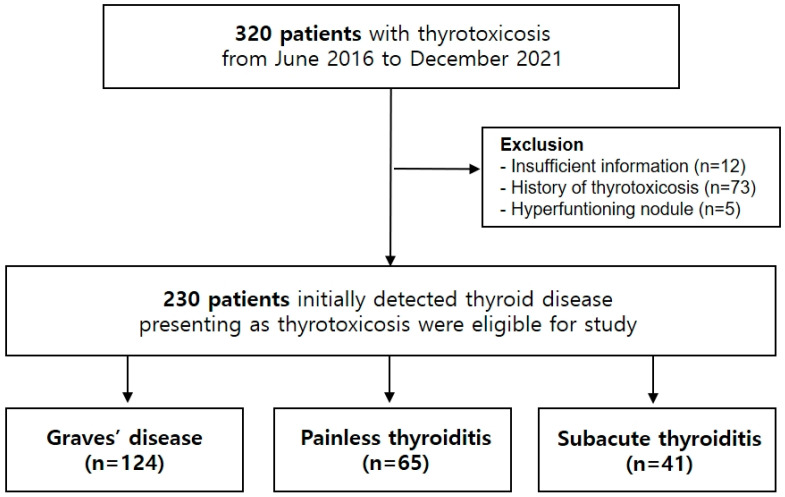
Flow chart of enrolled study subjects.

A total of 230 subjects with thyrotoxicosis was classified into three categories: Graves’ disease (*n* = 124), painless thyroiditis (*n* = 65), and subacute thyroiditis (*n* = 41) (Table 1). The median age of the study cohort was 47 years, and there was no difference in average age by disease. In all three disease categories, women had a higher prevalence than men. T3 and FT4 levels were significantly higher in Graves’ disease, ESR and CRP were significantly higher in subacute thyroiditis, and TBII and TSI were higher in Graves’ disease. TPOAb and TgAb were positive in 41.5% and 80.0%, respectively, of subjects with painless thyroiditis, but they were also elevated in about half of patients with Graves’ disease (59.7% and 50.4%). Clinical characteristics of the study cohort were described in Figure 1.

### 3.2. Predictive Values of Disease Specific Biomarkers

**Figure 2:**
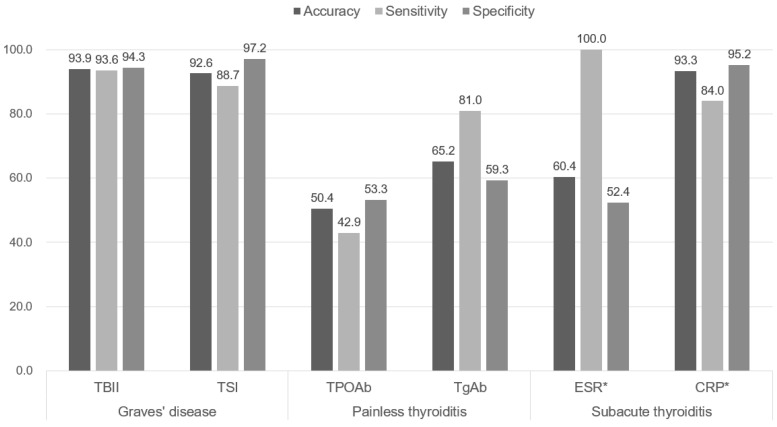
Accuracy, sensitivity, and specificity of biomarkers (%) for each disease are indicated by bar graphs. TBII, thyrotropin binding inhibitor immunoglobulin; TSI, thyroid stimulating immunoglobulin; TPOAb, anti-thyroid peroxidase antibody; TgAb, anti-thyroglobulin antibody; ESR, erythrocyte sedimentation rate; CRP, C-reactive protein * Available only in 149 patients.

TBII and TSI yield high accuracy for diagnosing Graves’ disease. ESR had high sensitivity for subacute thyroiditis, but specificity was low because of high false positive results. The values of sensitivity, specificity, and accuracy for each biomarker were shown in Figure 2.

### 3.3. Comparisons of Machine Learning Algorithms

**Figure 3:**
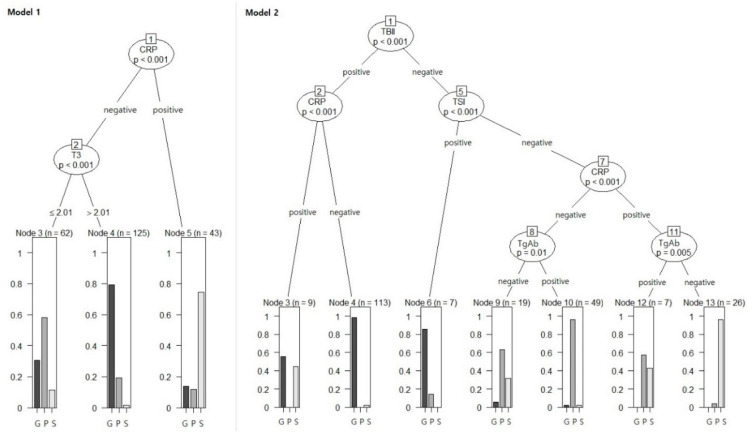
Decision tree models for Model 1 and Model 2. Node numbering for the decision trees presented inside the box. Classification according to the decision tree is described at the end of the tree, and the bar graph indicates the final diagnosis according to the clinical course. G, Graves’ disease; P, painless thyroiditis; S, subacute thyroiditis; T3, triiodothyronine; CRP, C-reactive protein; TBII, thyrotropin binding inhibitory immunoglobulin; TgAb, anti-thyroglobulin antibody.

**Figure 4:**
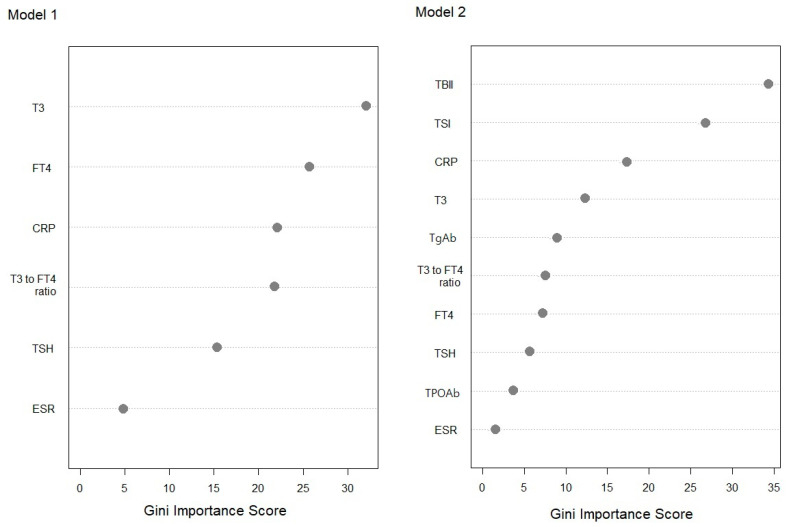
Variable importance plot for the random forest algorithm calculated using the impurity method for Model 1 and Model 2.

Diagnostic accuracy of seven machine learning algorithms constructed through different statistical methods were evaluated and described in Table 2. The algorithm with the highest accuracy for both Model 1 and Model 2 was RF. The significant variables were CRP and T3 in the decision tree of Model 1 and TBII, CRP, and TgAb in Model 2 (Figure 3), which were also the most significant variables in RF (Figure 4).

### 3.4. Clinical Validation Based on the Review of Medical Records

We compared the results of the RF algorithm with the standard diagnostic approach methods listed in Table 3. When Graves’ disease was diagnosed at the T3 level alone with a cut-off of 2.01 ng/mL, calculated from the CART model, and the accuracy was 75%. TBII showed higher accuracy than thyroid scan (94% vs. 82%). Model 2 with comprehensive information showed the highest accuracy, based on which we proposed that machine learning can be applied to differential diagnosis of thyrotoxicosis.

## 4. Discussion

We aimed to improve clinical practice by introducing a machine learning algorithm as an extension of statistical analysis for differential diagnosis. In our classifiers, accuracy ranged from 65–70% in Model 1 using only thyroid function test and inflammatory markers, and it ranged from 78–90% in Model 2 which also included thyroid autoantibody test results. Among various algorithms, RF showed the highest accuracy (Table 2).

Previous studies analyzed thyroid function test results according to age, sex, and etiology [9,10]. It has been suggested that T3, T4, and ratio of T3 to total thyroxine (T4) are high during early-onset Graves’ disease [11,12], which is characterized by an increase in thyroid hormone production [13]. In the results of this study, T3 level is the most significant variable among T3, FT4, and T3 to FT4 ratio in our classification models (Figure 3 and Figure 4). T3 has been suggested as a parameter to differentiate Graves’ disease from destruction-induced thyrotoxicosis by previous researchers [14], and it is recommended that T3 levels be monitored during initial treatment to observe the recovery speed during treatment [15]. The T3 cut-off to discriminate Graves’ disease was 2.01 ng/mL in this study, but when Graves’ disease was differentiated by T3 alone, the accuracy was as low as 75% (Table 3).

When thyroid antibody tests were added to the classification, TBII and TSI were high-accuracy tests for diagnosing Graves’ disease, and TBII was the most significant factor for classifying thyrotoxicosis. The second-generation TBII used in this study measures both stimulatory and inhibitory antibodies, and TSI could quantify the degree of stimulation by measuring intracellular cyclic adenosine monophosphate (cAMP) production [16], and the sensitivity of TSH is generally higher than that of TBII (Figure 2). Therefore, it was thought that TSI would be more useful in the evaluation of Graves’ disease, and there are studies showing that the association with eye symptoms [17] and predictive power of recurrence are higher than that of TBII [18]. However, TSI increases in proportion to TBII [19], and immunoglobulins that have a blocking role rather than stimulation may also affect thyroid function [20]. In addition, TSI is complex testing that takes a longer time to confirm the results, and the accuracy of TSI may vary depending on the methods and laboratory techniques [21,22]. There is also a controversy over high rates of false negative results for the pediatric patients [23] and uncertain diagnostic cut-off level in iodine-replete area [24]. TSI may be helpful in the diagnosis of Graves’ disease with false negative TBII results (Figure 3), but the variable importance of TBII was higher than that of TSI in the classification model (Figure 4). Therefore, we suggest that two assays are complementary to each other in the diagnosis of GD [25].

ESR has been widely used for diagnostic evaluation of subacute thyroiditis [26]. However, CRP performed better than ESR in this study (Figure 2). Although CRP is not used routinely to diagnose thyroid disease, previous studies have reported that CRP was positive in patients with subacute thyroiditis compared with those having other thyroid disorders [27]. Theoretically, CRP increases more rapidly than ESR in early-stage inflammatory disease, and it is often measured to evaluate inflammatory status in clinical situations. It might be more appropriate to use CRP rather than ESR to diagnose subacute thyroiditis, because ESR is a non-specific marker that is affected by patient age, sex, and renal function [28].

Thyroglobulin (Tg) is produced in the follicular cells of the thyroid gland and is synthesized as a precursor to thyroid hormone through the action of thyroid peroxidase (TPO). Autoantibodies for each substance—called anti-Tg-antibody (TgAb) and anti-TPO-antibody (TPOAb)—tend to be elevated in autoimmune thyroid disease, and are used in diagnostics [29]. Previous study presumed that TPOAb can be useful for screening because it has higher sensitivity than TgAb in thyroid dysfunction [30]. However, in this study, TgAb had superior accuracy compared to TPOAb for painless thyroiditis, and was confirmed a more significant classification factor (Figure 2, Figure 3 and Figure 4). Most cases of painless thyroiditis have a self-limiting course, but subsequent hypothyroidism or recurrent thyrotoxicosis after the first episode of thyrotoxicosis have been reported [31]. Therefore, the presence or absence of thyroid autoantibodies can help in diagnosing painless thyroiditis and may predict prognosis. This study suggests that TgAb is useful in autoimmune thyroid disease, which is consistent with the thoughts of previous researchers [32]. However, these antibodies are also increased in Grave’s disease, so they are not specific to painless thyroiditis [33]. In addition, the sensitivity and specificity of differential diagnoses can vary depending on case series, and clinicians need to pay attention to their interpretation.

With new developments in computational science, machine learning algorithms are being used as a way to implement artificial intelligence to support complex decision-making in many fields of medicine. Algorithms based on existing clinical data that predict diagnoses will facilitate clinical decision making and can be used for therapeutic interventions. Most previous studies that used artificial intelligence in clinical thyroid disease research focused on thyroid imaging findings. This study is the first to use machine learning in the initial diagnostic approach for patients with thyrotoxicosis. In thyrotoxicosis study, RF provided the highest accuracy, similar to the algorithm that provided the best accuracy in RF models of thyroid nodules in previous studies [34,35]. However, each algorithm may show different results depending on the characteristics of the data used, adjustment of modeling, statistical program, and detailed coding.

We employed seven well-known machine learning techniques for analysis. CART is the most widely used algorithm, and it forms a tree-shaped decision-making plot by selecting the variable with the lowest classification impurity and performing a binary split. RF is the model originated from CART, and it is called ‘forest’ because it operates by outputting average predictions from classifications determined by multiple decision trees formed in the training process [36]. For kNNs, LDAs, and SVMs, we map the data into space and then classify each entity. kNN works by grouping objects in order of the closest distance, hence the name ‘nearest neighbor’. LDA performs classification by finding the optimally classifiable line, and SVM by finding the hyperplane [37]. SVM using a multidimensional space is more accurate than LDA because it can predict data outside a normal distribution, but the algorithm is complex and may be time consuming. NB is a model using conditional probability in relation to Bayes’ theorem. The probability of predicting classification can be continuously improved by calculating changing probabilities by learning each individual as a new event using conditional probability. However, this model relies on a rather unrealistic assumption that each feature is probabilistically independent. This model provides a simple and quick judgment and can be used in the diagnosis of diseases on the assumption that the patient does not have more than one disease [38]. NN is a multi-layered model resembling the network structure of neurons in the human brain. Such a multi-layered structure requires adjustment and training of experienced technicians to ensure sufficient model performance, but is necessary when classifying more complex data such as audio or images [39].

A limitation of this study is that it was conducted with patients who live in an iodine-replete environment. In particular, the prevalence of thyrotoxicosis due to toxic nodule is high [40] and the false-negative rate of scan is low in the iodine-deficient region, and inputting the results of thyroid scintigraphy to machine learning models can help to increase the accuracy of the algorithm. Providing the patient’s symptoms—such as neck pain—as additional information to machine learning can also help to increase the accuracy of subacute thyroiditis diagnosis. Because this study analyzed a rather small number of patients using a machine learning tool, there is a risk of overfitting. As this study was conducted in a single center, the accuracy of our machine learning tool can be improved with inclusion of additional patients in the model, and external validation is needed for clinical application.

## 5. Conclusions

We investigated the laboratory findings of patients who presented with thyrotoxicosis with machine learning algorithms to perform differential diagnoses. The results of this study confirmed that early T3 level monitoring is useful for diagnosis of Graves’ disease at the first manifestation. Additionally, we found that TgAb was elevated in patients with autoimmune thyroid disease, which is consistent with previous studies; however, these are the first data which have been presented for thyrotoxicosis patients as far as we know. We suggest monitor the initial T3 and CRP levels with subsequent confirmation of TBII and TgAb in the differential diagnosis of thyrotoxicosis.

## Figures and Tables

**Table 1:** Baseline characteristics of the study cohort.

	Graves’ Disease (*n* = 124)	Painless Thyroiditis (*n* = 65)	Subacute Thyroiditis (*n* = 41)	*p*
Age years, mean (SD)	46.89 (14.81)	47.05 (11.30)	51.71 (13.46)	0.132
Female, number (%)	85 (67.7)	53 (81.5)	33 (80.5)	0.070
Thyroid function test				
TSH μIU/mL, mean (SD)	0.011 (0.033)	0.040 (0.159)	0.038 (0.091)	0.081
T3 ng/mL, mean (SD)	3.45 (1.75)	1.96 (0.83)	2.08 (0.70)	<0.001
FT4 ng/dL, mean (SD)	4.09 (2.15)	2.72 (1.09)	3.12 (1.58)	<0.001
Thyroid auto-antibodies				
TBII positive, number (%)	116 (93.5)	0 (0.0)	6 (14.6)	<0.001
TSI positive, number (%)	111 (89.5)	1 (1.5)	2 (4.7)	<0.001
TPOAb positive, number (%)	74 (59.7)	27 (41.5)	5 (11.6)	<0.001
TgAb positive, number (%)	62 (50.4)	52 (80.0)	5 (12.2)	<0.001
Inflammatory markers				
ESR mm/h, mean (SD)	13.39 (11.34)	14.61 (12.26)	66.08 (35.42)	<0.001
CRP mg/L, mean (SD)	1.36 (1.80)	1.93 (3.67)	35.46 (58.16)	<0.001

SD, standard deviation; TSH, thyroid stimulating hormone; T3, triiodothyronine; FT4, free thyroxine; TBII, TSH binding inhibitory immunoglobulin; TSI, thyroid stimulating immunoglobulin; TPOAb, anti-thyroid microsomal antibody; TgAb, anti-thyroglobulin antibody; ESR, erythrocyte sedimentation rate; CRP, C-reactive protein.

**Table 2:** Predicted value according to machine learning algorithm using initial blood test results (Model 1) and including thyroid antibody test results (Model 2).

Accuracy Classifier	Training Set (*n* = 161)				Test Set (*n* = 69)			
	Overall	G	P	S	Overall	G	P	S
**Model 1**								
**CART**	0.74	0.89	0.72	0.87	0.70	0.75	0.62	0.85
**RF**	0.80	0.82	0.77	0.87	0.70	0.69	0.58	0.85
**LDA**	0.74	0.79	0.72	0.87	0.70	0.74	0.60	0.85
**SVM**	0.76	0.80	0.73	0.87	0.65	0.69	0.51	0.85
**kNN**	0.75	0.73	0.65	0.81	0.67	0.78	0.63	0.70
**NB**	0.64	0.66	0.54	0.82	0.70	0.67	0.54	0.77
**NN**	0.74	0.79	0.71	0.87	0.68	0.69	0.58	0.80
**Model 2**								
**CART**	0.90	0.95	0.93	0.83	0.86	0.91	0.88	0.82
**RF**	0.98	0.98	0.99	0.97	0.90	0.96	0.90	0.86
**LDA**	0.91	0.96	0.93	0.86	0.87	0.93	0.88	0.86
**SVM**	0.92	0.97	0.93	0.87	0.87	0.93	0.88	0.86
**kNN**	0.82	0.86	0.80	0.89	0.78	0.79	0.75	0.89
**NB**	0.88	0.94	0.89	0.84	0.84	0.90	0.88	0.81
**NN**	0.91	0.95	0.95	0.84	0.88	0.92	0.95	0.86

G, Graves’ disease; P, painless thyroiditis; S, subacute thyroiditis; CART, classification and regression tree analysis; RF, random forest analysis; LDA, linear discriminant analysis; SVM, support vector machine; kNN, k-nearest neighbor; NB, naive Bayesian; NN, neural network.

**Table 3:** Clinical validation comparing the standard diagnostic approach and machine learning algorithms.

Diagnosed as Graves’ Disease	Graves’ Disease (*n* = 124)	Painless Thyroiditis (*n* = 65)	Subacute Thyroiditis (*n* = 41)	Accuracy for Graves’ Disease
T3 ^†^	101	18	17	0.75
TBII	116	0	6	0.94
Thyroid scan *	48/73	1/48	2/32	0.82
Initial ATD Prescription	79	11	3	0.74
RF Model 2	122	0	1	0.96

TBII, Thyrotropin binding inhibitor immunoglobulin; ATD, anti-thyroid drug; RF, random forest. ^†^ The cut-off level was 2.01 ng/mL, and it was defined by classification and decision tree models. * Available only in 153 patients.

## Data Availability

The data that support the findings of this study are not publicly available due to the information that could compromise the privacy of research participants but are available from the corresponding author (K.-H.B.) upon reasonable request.

## References

[1] Ross D.S., Burch H.B., Cooper D.S., Greenlee M.C., Laurberg P., Maia A.L., Rivkees S.A., Samuels M., Sosa J.A., Stan M.N. (2016). 2016 American thyroid association guidelines for diagnosis and management of hyperthyroidism and other causes of thyrotoxicosis. Thyroid.

[2] Kahaly G.J., Bartalena L., Hegedus L., Leenhardt L., Poppe K., Pearce S.H. (2018). 2018 European thyroid association guideline for the management of graves’ hyperthyroidism. Eur. Thyroid J..

[3] Nikolai T.F., Brosseau J., Kettrick M.A., Roberts R., Beltaos E. (1980). Lymphocytic thyroiditis with spontaneously resolving hyperthyroidism (silent thyroiditis). Arch. Intern. Med..

[4] Fatourechi V., Aniszewski J.P., Fatourechi G.Z., Atkinson E.J., Jacobsen S.J. (2003). Clinical features and outcome of subacute thyroiditis in an incidence cohort: Olmsted County, Minnesota, study. J. Clin. Endocrinol. Metab..

[5] Barbesino G., Tomer Y. (2013). Clinical review: Clinical utility of TSH receptor antibodies. J. Clin. Endocrinol. Metab..

[6] Perdomo C.M., García-Goñi M., Sancho L., Paricio J., Lozano M.D., de la Higuera M., Currás M., Arbizu J., Galofré J.C. (2021). Evaluation of the role of thyroid scintigraphy in the differential diagnosis of thyrotoxicosis. Clin. Endocrinol..

[7] Grayson R.R. (1960). Factors which influence the radioactive iodine thyroidal uptake test. Am. J. Med..

[8] Schulz S.L., Seeberger U., Hengstmann J.H. (2003). Color Doppler sonography in hypothyroidism. Eur. J. Ultrasound.

[9] Reinwein D., Benker G., König M.P., Pinchera A., Schatz H., Schleusener A. (1988). The different types of hyperthyroidism in Europe. Results of a prospective survey of 924 patients. J. Endocrinol. Investig..

[10] Yanai H., Hakoshima M., Katsuyama H. (2019). Differences in clinical and laboratory findings among graves’ disease, painless thyroiditis and subacute thyroiditis patients with hyperthyroidism. J. Endocrinol. Metab..

[11] Amino N., Yabu Y., Miki T., Morimoto S., Kumahara Y., Mori H., Iwatani Y., Nishi K., Nakatani K., Miyai K. (1981). Serum ratio of triiodothyronine to thyroxine, and thyroxine-binding globulin and calcitonin concentrations in Graves’ disease and destruction-induced thyrotoxicosis. J. Clin. Endocrinol. Metab..

[12] Carlé A., Knudsen N., Pedersen I.B., Perrild H., Ovesen L., Rasmussen L.B., Laurberg P. (2013). Determinants of serum T4 and T3 at the time of diagnosis in nosological types of thyrotoxicosis: A population-based study. Eur. J. Endocrinol..

[13] Woeber K.A. (2006). Triiodothyronine production in Graves’ hyperthyroidism. Thyroid.

[14] Izumi Y., Hidaka Y., Tada H., Takano T., Kashiwai T., Tatsumi K.I., Ichihara K., Amino N. (2002). Simple and practical parameters for differentiation between destruction-induced thyrotoxicosis and Graves’ thyrotoxicosis. Clin. Endocrinol..

[15] Chen J.J., Ladenson P.W. (1986). Discordant hypothyroxinemia and hypertriiodothyroninemia in treated patients with hyperthyroid Graves’ disease. J. Clin. Endocrinol. Metab..

[16] Evans C., Morgenthaler N.G., Lee S., Llewellyn D.H., Clifton-Bligh R., John R., Lazarus J.H., Chatterjee V.K., Ludgate M. (1999). Development of a luminescent bioassay for thyroid stimulating antibodies. J. Clin. Endocrinol. Metab..

[17] Lytton S.D., Ponto K.A., Kanitz M., Matheis N., Kohn L.D., Kahaly G.J. (2010). A novel thyroid stimulating immunoglobulin bioassay is a functional indicator of activity and severity of Graves’ orbitopathy. J. Clin. Endocrinol. Metab..

[18] Giuliani C., Cerrone D., Harii N., Thornton M., Kohn L.D., Dagia N.M., Bucci I., Carpentieri M., Di Nenno B., Di Blasio A. (2012). A TSHR-LH/CGR chimera that measures functional thyroid-stimulating autoantibodies (TSAb) can predict remission or recurrence in Graves’ patients undergoing antithyroid drug (ATD) treatment. J. Clin. Endocrinol. Metab..

[19] Cheng X., Chai X., Ma C., Jia Q., Zhao H., Dong Z., Zhang Z., Hu Y., Song A., Yang G. (2021). Clinical diagnostic performance of a fully automated TSI immunoassay vs. that of an automated anti-TSHR immunoassay for Graves’ disease: A Chinese multicenter study. Endocrine.

[20] McLachlan S.M., Rapoport B. (2013). Thyrotropin-blocking autoantibodies and thyroid-stimulating autoantibodies: Potential mechanisms involved in the pendulum swinging from hypothyroidism to hyperthyroidism or vice versa. Thyroid.

[21] Lytton S.D., Kahaly G.J. (2010). Bioassays for TSH-receptor autoantibodies: An update. Autoimmun. Rev..

[22] Kiaei D., Molinaro R. (2020). A negative thyretain TSI bioassay result does not exclude the possibility of the presence of TSI. Horm. Metab. Res..

[23] Rahhal S.N., Eugster E.A. (2008). Thyroid stimulating immunoglobulin is often negative in children with Graves’ disease. J. Pediatr. Endocrinol. Metab..

[24] Lee J.I., Jang H.W., Kim S.K., Choi J.Y., Kim J.Y., Hur K.Y., Kim J.H., Min Y.K., Chung J.H., Kim S.W. (2011). Diagnostic value of a chimeric TSH receptor (Mc4)-based bioassay for Graves’ disease. Korean J. Intern. Med..

[25] Takasu N., Oshiro C., Akamine H., Komiya I., Nagata A., Sato Y., Yoshimura H., Ito K. (1997). Thyroid-stimulating antibody and TSH-binding inhibitor immunoglobulin in 277 Graves’ patients and in 686 normal subjects. J. Endocrinol. Investig..

[26] Benbassat C.A., Olchovsky D., Tsvetov G., Shimon I. (2007). Subacute thyroiditis: Clinical characteristics and treatment outcome in fifty-six consecutive patients diagnosed between 1999 and 2005. J. Endocrinol. Investig..

[27] Pearce E.N., Bogazzi F., Martino E., Brogioni S., Pardini E., Pellegrini G., Parkes A.B., Lazarus J.H., Pinchera A., Braverman L.E. (2003). The prevalence of elevated serum C-reactive protein levels in inflammatory and noninflammatory thyroid disease. Thyroid.

[28] Osei-Bimpong A., Meek J.H., Lewis S.M. (2007). ESR or CRP? A comparison of their clinical utility. Hematology.

[29] Gilmour J., Brownlee Y., Foster P., Geekie C., Kelly P., Robertson S., Wade E., Braun H.B., Staub U., Michel G. (2000). The quantitative measurement of autoantibodies to thyroglobulin and thyroid peroxidase by automated microparticle based immunoassays in Hashimoto’s disease, Graves’ disease and a follow-up study on postpartum thyroid disease. Clin. Lab..

[30] Frohlich E., Wahl R. (2017). Thyroid Autoimmunity: Role of Anti-thyroid Antibodies in Thyroid and Extra-Thyroidal Diseases. Front. Immunol..

[31] Ohye H. (2008). Recurrent severe painless thyroiditis requiring multiple treatments with radioactive iodine. Thyroid.

[32] Nishihara E., Amino N., Kudo T., Ito M., Fukata S., Nishikawa M., Nakamura H., Miyauchi A. (2017). Comparison of thyroglobulin and thyroid peroxidase antibodies measured by five different kits in autoimmune thyroid diseases. Endocr. J..

[33] Choi Y.M., Kwak M.K., Hong S.M., Hong E.G. (2019). Changes in Thyroid Peroxidase and Thyroglobulin Antibodies Might Be Associated with Graves’ Disease Relapse after Antithyroid Drug Therapy. Endocrinol. Metab..

[34] Zhang B., Tian J., Pei S., Chen Y., He X., Dong Y., Zhang L., Mo X., Huang W., Cong S. (2019). Machine learning-assisted system for thyroid nodule diagnosis. Thyroid.

[35] Zhao C.K., Ren T.T., Yin Y.F., Shi H., Wang H.X., Zhou B.Y., Wang X.R., Li X., Zhang Y.F., Liu C. (2021). A comparative analysis of two machine learning-based diagnostic patterns with thyroid imaging reporting and data system for thyroid nodules: Diagnostic performance and unnecessary biopsy rate. Thyroid.

[36] Breiman L. (2001). Random forests. Mach. Learn..

[37] Cortes C. (1995). Suppor-vector network. Mach. Learn..

[38] Hand D.J., Yu K. (2001). Idiot’s bayes: Not so stupid after all?. Int. Stat. Rev..

[39] Yadav S.S., Jadhav S.M. (2019). Deep convolutional neural network based medical image classification for disease diagnosis. J. Big Data.

[40] Laurberg P., Cerqueira C., Ovesen L., Rasmussen L.B., Perrild H., Andersen S., Pedersen I.B., Carlé A. (2010). Iodine intake as a determinant of thyroid disorders in populations. Best Pract. Res. Clin. Endocrinol. Metab..

